# Manual of the Principles and Practice of Operative Surgery

**Published:** 1879-08

**Authors:** 


MANUAL OF THE PRINCIPLES AND PRACTICE OF OPERATIVE SURGERY. By Stephen Smith, A.M., M.D., Surgeon to Bellevue and St. Vincent Hospitals, New York. Houghton, Osgood & Co., publishers, Boston. 1879.

This is a closely printed book of six hundred and sixty-odd pages, and contains much that is instructive and interesting. We have long admired and been benefitted by the work of the same character of Henry H. Smith, and hesitate not to say that the one before us is not only equal to, but surpasses the older work. The principles with the most approved practice are given to the fullest extent, and we recommend the book as the best we have ever seen on operative surgery Stimson's manual of operative surgery, published at Philadelphia, is very good, but not so voluminous as the one before us. We think works of this character are calculated to work much benefit, and are satisfied that time spent in reading them will not be lost. Each surgical disease and injury is treated of explicitly and in a systematic order, founded upon the particular part affected.

